# Non-Lethal Endotoxin Injection: A Rat Model of Hypercoagulability

**DOI:** 10.1371/journal.pone.0169976

**Published:** 2017-01-12

**Authors:** Marjory B. Brooks, James R. Turk, Abraham Guerrero, Padma K. Narayanan, John P. Nolan, Elizabeth G. Besteman, Dennis W. Wilson, Roberta A. Thomas, Cindy E. Fishman, Karol L. Thompson, Heidrun Ellinger-Ziegelbauer, Jennifer B. Pierson, April Paulman, Alan Y. Chiang, Albert E. Schultze

**Affiliations:** 1 Comparative Coagulation Section, Department of Population Medicine and Diagnostic Sciences, Cornell University, Ithaca, NY, United States of America; 2 Comparative Biology and Safety Sciences, Amgen Inc., Thousand Oaks, CA, United States of America; 3 Scintillon Institute, San Diego, CA, United States of America; 4 Department of Pathology, Safety Assessment and Laboratory Animal Resources, Merck Research Laboratories, West Point, PA, United States of America; 5 Department of Pathology Microbiology and Immunology, School of Veterinary Medicine, University of California-Davis, Davis, CA, United States of America; 6 GlaxoSmithKline, Research and Development, King of Prussia, Pennsylvania, United States of America; 7 Center for Drug Evaluation and Research, United States Food and Drug Administration, Silver Spring, MD, United States of America; 8 Bayer Pharma AG, Aprather Weg,Wuppertal, Germany; 9 Health and Environmental Sciences Institute, Suite, Washington, DC, United States of America; 10 Department of Pathology, Covance Laboratories, Greenfield, IN, United States of America; 11 Global Statistical Sciences, Lilly Research Laboratories, Indianapolis, IN, United States of America; 12 Pathology Department, Lilly Research Laboratories, Lilly Corporate Center, Indianapolis, IN, United States of America; Maastricht University Medical Center, NETHERLANDS

## Abstract

Systemic inflammation co-activates coagulation, which unchecked culminates in a lethal syndrome of multi-organ microvascular thrombosis known as disseminated intravascular coagulation (DIC). We studied an endotoxin-induced inflammatory state in rats to identify biomarkers of hemostatic imbalance favoring hypercoagulability. Intraperitoneal injection of LPS at 15 mg/kg body weight resulted in peripheral leukopenia and widespread neutrophilic sequestration characteristic of an acute systemic inflammatory response. Early indicators of hemostatic pathway activation developed within 4 hours, including increased circulating concentrations of procoagulant extracellular vesicles (EVs), EVs expressing endothelial cell and platelet membrane markers, and high concentration of soluble intercellular adhesion molecule-1 (sICAM-1), plasminogen activator inhibitor-1 (PAI-1), and D-dimers. Inflammation persisted throughout the 48-hour observation period; however, increases were found in a subset of serum microRNA (miRNA) that coincided with gradual resolution of hemostatic protein abnormalities and reduction in EV counts. Dose-adjusted LPS treatment in rats provides a time-course model to develop biomarker profiles reflecting procoagulant imbalance and rebalance under inflammatory conditions.

## Introduction

Innate immunity represents the body’s earliest defense against pathogen invasion. The integral components of this response include phagocytic and cytotoxic leukocytes and the proteolytic enzymes of the complement cascade [1]. Platelets and coagulation factors share evolutionary origins with these cellular and humoral reactants, and the interrelatedness of hemostasis and immunity is becoming increasingly apparent [2, 3]. The adverse effects of co-activation of these pathways manifest as thrombosis and multi-organ failure in patients with infectious and non-infectious inflammatory disease [4]. However, the mechanisms that differentiate protective versus pathologic hemostasis in an inflammatory milieu remain ill-defined.

Infusion of bacterial endotoxin or lipopolysaccharide (LPS) is often used to model the inflammatory state induced by bacterial sepsis [5–7]. Endotoxin activates leukocytes and endothelial cells, and with increasing dosage leads to a systemic inflammatory response syndrome (SIRS) characterized by loss of vasomotor control and increased vascular permeability. Mortality in severe sepsis often results from dysregulation of the host response rather than direct tissue injury by the infectious agent [4, 8]. Systemic activation of coagulation occurs simultaneously with SIRS in the LPS model and uncontrolled coagulation is recognized as the cause of death in many patients succumbing to sepsis. This process, referred to as disseminated intravascular coagulation (DIC), represents a dynamic state of hemostatic imbalance that may progress to widespread microvascular fibrin deposition, occlusive thrombosis, hemorrhage, and tissue necrosis [7–9]. Overt DIC is recognized by clinical criteria of thrombocytopenia, prolonged clotting times, depletion of fibrinogen and antithrombin, and high concentration of circulating fibrin degradation products [10]. In contrast, early subclinical, or non-overt DIC has no defined laboratory or histologic abnormalities, and no predictive tests to differentiate patients who re-establish effective hemostatic balance from those who progress to overt DIC and death [9, 10].

Intravenous administration of a high dose of LPS induces mortality in rats due to fulminant, overt DIC [6, 7]. We undertook refinement of this lethal dosage to identify biomarkers of procoagulant imbalance preceding microvascular thrombosis, and features of hemostatic rebalance and recovery. Characterization of the interrelationship between inflammation and coagulation is also relevant for common conditions such as atherothrombosis and diabetes, now recognized as chronic inflammatory states [11, 12]. Coincident with global increases in prevalence of these disorders are more frequent reports of drug-induced thrombotic events [13, 14]. Our recent survey of preclinical drug development revealed a critical gap in early detection of incipient thrombosis in the absence of lethal and grossly visible thrombi on post mortem examination in animal models [15]. In this time course study of sub-lethal LPS administration in rats, we combined comprehensive clinicopathologic tests and detailed histologic review with newer techniques analyzing extracellular vesicles and miRNA, to identify circulating biomarkers with potential applications as screening tests of procoagulant imbalance.

We hypothesized that the use of low dose LPS would perturb, but not overwhelm, hemostatic balance such that serial monitoring over time would reveal changes in circulating biomarkers that reflected early activation of inflammation and coagulation and a subsequent homeostatic response.

## Materials and Methods

### Animals and Experimental Design

Animal studies were performed at Covance Laboratories, Inc. (Greenfield, IN, USA) which is accredited by the AAALAC International. The Institutional Animal Care and Use Committee (IACUC) at Covance Laboratories reviewed and approved the study (protocol 8324813, approved December, 2014). The study was conducted in compliance with the Guide for the Care and Use of Laboratory Animals and the Office of Laboratory Animal Welfare. Male Wistar rats (N = 50; 268 to 330 grams body weight) were obtained from Charles River Laboratories (Portage, MI, USA). Rats were housed in groups of 2 to 3 under controlled twelve-hour light: dark cycle, temperature (68–79°F), and humidity (30%-70%), and fed a commercial diet (Harlan Teklad 2014 diet, Harlan Teklad, Madison, WI, USA) with filtered tap water ad libitum.

The rats were randomized to receive intraperitoneal injections of either vehicle control (0.9% sodium chloride, Vedco, Saint Joseph, MO, USA) or 15 mg/kg LPS [(*Escherichia coli* LPS, 15 mg/mL), Calbiochem, EMD Biosciences San Diego, CA, USA]. At 1, 4, 8, 24, or 48 hours post-LPS or saline injection (*Table 1*), paired treatment cohorts of 5 rats per group were anesthetized, blood was collected, the animals were humanely euthanized, and tissue collection occurred.

**Table 1:** Experimental design and time-points analyzed.

Treatment Group	Euthanasia (Hours post-treatment)
Control (0 mg/kg)	1
	4
	8
	24
	48
LPS (15 mg/kg)	1
	4
	8
	24
	48

The designated sampling time-points are given for each group treated intraperitoneally with either vehicle control (saline) or LPS (15 mg/kg). n = 5/time-point/group.

### In-life Observations, Blood Collection, Processing

Clinical observations were made prior to dosing, 1 hour and 3 to 6 hours after dosing, and prior to necropsy on Day 2. Detailed observations were recorded and experienced technical staff evaluated all animals on study for evidence of pain or suffering that might warrant intervention.

Isoflurane inhalation anesthesia was used for terminal blood collection directly from the abdominal aorta. Blood for evaluation of soluble E-selectin (sE-selectin) and soluble intercellular adhesion molecule-1(sICAM-1) concentrations and miRNA analysis was drawn into glass tubes (2 mL Monoject, Covidien, Mansfield, MA, USA), held at room temperature to clot, and serum was frozen at ≤-60°C. Blood for hemograms was drawn into K_3_-EDTA tubes (2 mL Monoject, 7.5% EDTA solution, Covidien, Mansfield, MA, USA). Blood for coagulation testing, nanoparticle analysis, and platelet flow cytometry was drawn into citrate tubes (2 mL, 3.2% sodium citrate, BD, Franklin Lakes, NJ, USA). Approximately 300 μL of citrated whole blood was reserved for flow cytometry and the remainder was spun twice (2000 g x 15 minutes). Plasma aliquots were assayed immediately for prothrombin time (PT), activated partial thromboplastin time (APTT), and fibrinogen, or stored frozen at ≤-60°C for subsequent nanoparticle analyses and determination of antithrombin activity (AT), and concentrations of D-dimer and plasminogen activator inhibitor 1 (PAI–1).

### Complete Blood Counts and Coagulation Testing

The ADVIA 120 Hematology System with Multispecies Software (Version 3.1, Siemens Medical Solutions, Norwood, MA, USA) and Siemens reagents were used for automatic or calculated cell counts, hemoglobin concentration, mean corpuscular volume, hematocrit, mean corpuscular hemoglobin, and mean corpuscular hemoglobin concentration. Blood cell morphology and differential leukocyte counts were confirmed by review of Wright-Giemsa stained (ADVIA S60 Auto Slide Stainer, Siemens Medical Solutions) blood smears.

The PT, APTT, and fibrinogen were measured using an automated coagulation instrument (STA Compact, American Bio Products, Parsippany, NJ, USA).

### Hemostatic Protein Analyses (D-Dimer, Antithrombin, PAI-1)

Plasma D dimer concentration was measured using an anti-human ELISA (Asserachrom® D-Di, Diagnostica Stago, Parsippany, NJ, USA), PAI-1 was measured using an ELISA (Rat PAI-1 Activity ELISA Kit, Catalog #RPAIKT, Molecular Innovations, Novi, MI, USA) configured to detect functional PAI-1 based on its binding to urokinase-coated microtiter plate wells, and antithrombin (AT) was measured using a chromogenic substrate kit (Stachrom® ATIII, Diagnostica Stago, Parsippany, NJ, USA) based on inhibition of bovine thrombin (anti-IIa assay).

### Soluble E-Selectin and Soluble Intercellular Adhesion Molecule-1

Soluble forms of E-Selectin and ICAM-1 were measured with Milliplex™ MAP Rat Cardiovascular Disease Panel 2 (EMD Millipore, Billerica, MA, USA) a Luminex-based multiplex methodology.

### Flow Cytometry

Duplicate aliquots of citrated blood from test and control rats were incubated with fluorescein labeled fibrinogen (DAKO FO111) and phycoerythrin labeled anti-human CD62P (Serotec MCA2419), and then fixed and analyzed (Beckman Coulter FC500, Beckman Coulter, Miami, FL, USA). For negative and positive assay controls, blood was collected from a single stock rat on each assay date and preincubated with either a platelet inhibitory agent (0.25 units apyrase) or platelet agonist (10 μM ADP). The platelet population was identified by light scatter properties with collection to 10,000 platelet-gated events or a maximum acquisition time of 120 seconds.

### Extracellular vesicle (EV) analysis

The EV content was measured by vesicle flow cytometry (VFC) using a membrane-intercalating dye and a custom high sensitivity flow cytometer, as previously described [16]. Each sample was stained with a fluorogenic membrane probe, di-8ANEPPS, and one each of four DyLight488-labeled surface markers: annexin V (A. Brisson, University of Bordeaux, France), anti-CD42d ([RPM-4] BD Biosciences, San Jose, CA), anti-CD54 (CD54 clone [1A29] BD Biosciences), or anti-CD106 (CD106 clone [MR106], Biolegend, San Diego, CA).

Samples were serially diluted in filtered physiologic buffered saline (PBS) for di-8-ANEPPS labeling (100 nM, 2 hours, room temperature) to optimize probe to membrane ratios for quantitative analysis. Labeled aliquots were then incubated with annexin V or antibodies (100 nM, 1 hour, room temperature) before 200-fold dilution in filtered PBS.

Green (DyLight 488) and red (di-8-ANEPPS) fluorescence was collected through a 525/40 bandpass or a 600 LP, respectively. Vesicle detection was triggered on di-8-ANEPPS fluorescence and samples were measured for 1 minute. The sample volumetric flow rate was determined by measuring beads (5.23 μm AccuCount, Spherotech, Lake Forest, IL). Marker positivity threshold was based on background from free dye in the sample stream determined by software-triggering of the instrument. Green fluorescence intensity was calibrated in mean equivalent molecules of fluorescein (MESF) using multi-intensity reference beads (Rainbow, Spherotech) that had been cross-calibrated with fluorescein intensity standard beads (Quantum FITC MESF, Bangs Laboratories, Fishers, IN).

Nanoparticle Tracking Analysis (NTA) was performed on PBS diluted samples loaded into the chamber of a Nanosight LM-20 (Malvern Instruments, Malvern, UK) equipped with a 532 nm laser and a CCD camera. The optimal camera level (setting: 15) and threshold (setting: 2) were established as previously described[17]. Five movies of 60 seconds each were recorded and analyzed for each sample to obtain average histograms and mean diameters.

### miRNA Isolation and Analysis

To identify miRNAs as potential circulating markers indicating procoagulant imbalances in our model study, total RNA including small RNAs was isolated from rat serum and then profiled on Taqman Low Density Array (TLDA) Rodent miRNA Array A. This array represents 378 unique rodent miRNAs and several small RNAs on a micro-fluidic card preloaded with corresponding primers and probes allowing for quantitative PCR based on Taqman chemistry.

Briefly, total RNA was isolated from 200 ul rat serum (one sample per animal) using the mirVana™ miRNA Isolation Kit (LifeTechnologies, Carlsbad, CA, USA) as previously described.[18] Total RNA concentrations were estimated using a NanoDrop Spectrophotometer (ThermoScientific, Wilmington, DE) then quantified using the RiboGreen Quantification Kit Low Range Assay (Molecular Probes, Eugene, OR). cDNA was synthesized from approximately 3ng total RNA from each sample using the Taqman® miRNA Reverse Transcription Kit (Life Technologies) and reverse transcription (RT) primers (Taqman® miRNA Array A Megaplex pool, Life Technologies) specific for Taqman Low Density Array (TLDA) Rodent miRNA Array A. Equal volumes of cDNA were added to PreAmp reactions (Taqman® PreAmp Master Mix) and a PreAmp primer pool specific for TaqMan® Array Rodent miRNA A Card v3.0. Upon completion of the PreAmp reactions, miRNA was amplified and quantified using one Rodent miRNA TLDA per sample on a Viia7 (Life Technologies) real time PCR instrument.

miRNA data analysis was performed using Expression Suite Software v1.0.3 (LifeTechnologies) then C_T_ values were exported to Excel and the ΔΔC_T_ method was used as described in Applied Biosystems User Bulletin 2: ABI Prism 7900 Sequence Detection System (Foster City, CA). Briefly, ΔC_T_ values were obtained by subtracting the reference gene (mmu-miR-15b) C_T_ from the C_T_ for each miRNA of interest. ΔΔC_T_ values were obtained by subtracting the average control group ΔC_T_ value from each of the individual ΔC_T_ values. Input values were generated by using the formula (2^-(ΔΔ^C_T_^)^) for each sample for each miRNA. Group average input values were calculated for each miRNA, then calibrated to the time-matched Control Group Average input for each miRNA (set at “1”), referred to as Relative Quantity (RQ). A student’s t-test using 2-tailed, unequal variance was used to evaluate differences for each miRNA between the normalized control and treated input values [19]. Our objective was to identify potential biomarkers of hypercoagulability induced by LPS, thus only increases from control were considered. Next, inclusive criteria to identify candidate biomarkers were used, which were a group mean fold-change from time-matched controls greater than 2, with p-value of ≤ 0.05 for at least 1 of 5 time-points. Subsequently, Ingenuity pathway analysis (IPA, Ingenuity Systems, www.ingenuity.com) was employed to reveal direct connections previously described among the miRNAs found increased in our study, the hemostatic and adhesive proteins we measured, and an LPS stimulus.

### Necropsy and Histopathologic Examination

Representative samples of brain, heart, kidney, liver, lung, mesentery, and spleen, and macroscopic lesions were collected at necropsy, fixed in 10% neutral buffered formalin, and embedded in paraffin for sectioning and hematoxylin and eosin staining. Microscopic examinations and pathology data review were performed independently by two board certified veterinary pathologists.

The heart, lungs, and mesentery were processed to optimize vascular histopathologic examination. An intratracheal catheter was inserted prior to en bloc removal of the heart and lungs. The base of the heart was then ligated to maintain proper filling of the pulmonary arteries before separation from the lungs and fixation. The lungs were inflated to 20 cm H_2_O by instillation of formalin through the catheter and fixed for one hour with exchange of formalin as needed. After fixation, each lung lobe was sectioned from the hilus to the distal pleural border, parallel to the main stem bronchus and perpendicular to the flat surface of the lobe, and sections were then embedded in paraffin blocks.

After dissection of the entire mesentery and removal of mesenteric lymph nodes, the mesentery was laid flat on brown paper, fixed in 10% neutral buffered formalin, and cut in one third sections with the cut surface placed so the mesenteric vessels were perpendicular to the block face.

### Statistical Analysis

Quantitative results for hematology, coagulation, soluble E-Selectin and ICAM-1, platelet P-Selectin and bound fibrinogen, and EV parameters were analyzed using a two-factor analysis of variance with PROC MIXED in SAS 9.2. Factors in the model included treatment, time and the interaction of treatment-by-time. The LPS-induced treatment effects were evaluated by contrast t-tests for each time point at the 0.05 significance level (2-sided). Shapiro-Wilk’s test [20] for normality was performed at the 0.01 significance level and Levene’s test [21] for homogeneity of variance was performed at the 0.01 significance level to assist in interpretation of treatment effects. If either Shapiro-Wilk’s test or Levene’s test was statistically significant, the data were rank-transformed and the LPS induced treatments evaluated by Wilcoxon rank-sum tests for each time point at the 0.05 significance level (2-sided). No multiplicity adjustment was made for the multiple comparisons at different time points.

## Results

### Clinical Signs, Hemogram Changes, and Platelet Activation Markers

No mortality occurred during the study. Rats given control vehicle (sterile saline) had no clinical abnormalities at any time. Few rats treated with LPS had non-specific signs of systemic illness. Squinting and wet and soiled fur began within 4 to 8 hours after LPS administration and persisted until study termination. Decreased activity, lacrimation, hunched posture and rough haircoat were observed at 24 and/or 48 hours in rats treated with LPS. In addition, transient episodes of soft and/or watery feces occurred in a few LPS-treated rats within 4 hours after dose administration (Table 2).

**Table 2:** Clinical observations in LPS-treated animals.

LPS Dose	15 mg/kg				
Observation Time Point	1 hr	4 hr	8 hr	24 hr	48 hr
Number of Treated Rats Sacrificed after Observation	5	5	5	5	5
Number of Treated Rats Remaining under Observation	25	20	15	10	5
Clinical Observations	Number of Animals Affected (% Treated Rats Remaining)				
Decreased activity	0	0	0	3 (30%)	0
Squinting	0	3 (15%)	4 (27%)	4 (40%)	4 (80%)
Lacrimation	0	0	0	2 (20%)	2 (40%)
Hunched posture	0	0	2 (13%)	2 (20%)	3 (60%)
Rough haircoat	0	0	1 (7%)	2 (20%)	2 (40%)
Soiling, perianal	0	3 (15%)	1 (7%)	3 (30%)	4 (80%)
Soiling, ventral abdomen	0	0	0	4 (40%)	0
Soiling, forepaws	0	0	0	3 (30%)	0
Soiling, nose	0	0	0	1 (10%)	0
Soiling, eyelids	0	0	0	3 (30%)	0
Wetness, perianal	0	0	0	1 (10%)	0
Wetness, urogenital	0	0	0	1 (10%)	0
Wetness, ventral abdomen	0	0	0	0	2 (40%)
Feces, soft	0	3 (15%)	0	0	0
Feces, watery	0	0	0	2 (20%)	0

none of the rats given saline vehicle had abnormal clinical signs at any time point. Incidence of clinical observations in LPS (15 mg/kg) treated rats.

**Fig 1:**
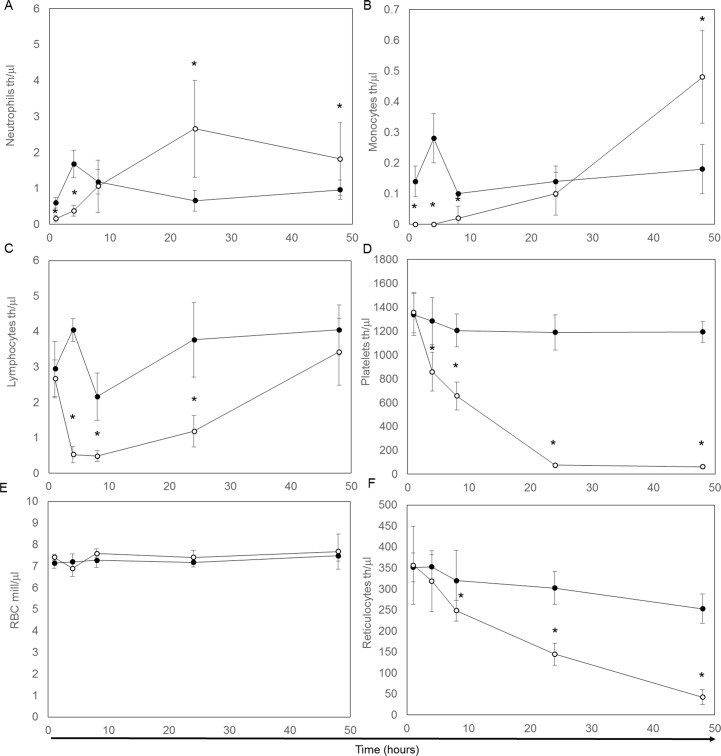
Hemogram cell counts over a 48 hour period. Individual cell counts from both control (●) and LPS (o) for neutrophils (A), monocytes (B), lymphocytes (C), platelets (D), red blood cells (E), and reticulocytes (F). Presented are means and standard deviation for five animals at each time point. * indicates a significant (P<0.05) difference between the control and LPS-treated groups.

Hemogram changes characteristic of a peracute inflammatory response to LPS were evident within 1 hour. (*Fig 1 and Table 3)*. A rapid decrease in absolute neutrophil and monocyte counts developed in the first hour, followed by a rebound increase above that of controls by 24 and 48 hours, respectively (*Fig 1A and 1B*). Morphologic evidence of “toxic” changes in neutrophils including cytoplasmic granulation and basophilia were also noted at the 24 and 48 hour timepoints. Lymphocyte counts decreased, with nadir at 4 to 8 hours post-administration and then a gradual increase to control values by 48 hours (*Fig 1C*). Platelet counts also demonstrated a progressive decrease with severe thrombocytopenia by 24 hours that persisted to 48 hours (*Fig 1D).* A slight increase in hematocrit, compatible with mild dehydration, developed at 1 hour (*Table 3*), however red blood cell counts remained stable and did not differ significantly from controls *(Fig 1E).* In contrast, reticulocyte counts gradually fell and were significantly lower than controls by 8 hours and for the remainder of the study (*Fig 1F*).

**Table 3:** Complete blood count results from rats given saline vehicle or LPS.

Parameter	Treatment	Time Point (Hours)					Reference Interval
		1	4	8	**24**	**48**	
**Reticulocyte (th/μL)**	**Vehicle Control**	351.5 ± 34.6	352.8 ± 29.0	319.8 ± 72.1	302.1 ± 39.4	252.8 ± 34.7	152.3–381.5
	**LPS**	356.1 ± 92.6	318.6 ± 72.2	248.2 ± 24.8*	144.5 ± 26.0*	42.4 ± 18.0*	
**Erythrocyte (mill/μL)**	**Vehicle Control**	7.14 ± 0.24	7.19 ± 0.38	7.27 ± 0.33	7.17 ± 0.21	7.47 ± 0.25	7.27–9.65
	**LPS**	7.40 ± 0.14	6.89 ± 0.37	7.58 ± 0.21	7.40 ± 0.33	7.67 ± 0.81	
**Hemoglobin (g/dL)**	**Vehicle Control**	13.7 ± 0.4	14.2 ± 0.6	14.1 ± 0.5	13.8 ± 0.4	14.1 ± 0.4	13.7–17.6
	**LPS**	14.5 ± 0.1*	13.4 ± 0.4*	14.5 ± 0.4	14.3 ± 0.5	14.7 ± 1.2	
**Hematocrit (%)**	**Vehicle Control**	43.6 ± 1.1	45.1 ± 1.5	44.5 ± 1.6	44.1 ± 1.5	44.9 ± 1.0	39.6–52.5
	**LPS**	46.1 ± 0.8*	42.5 ± 1.2*	45.4 ± 1.4	44.2 ± 2.0	45.6 ± 4.1	
**MCV (fL)**	**Vehicle Control**	61.1 ± 2.3	62.7 ± 1.8	61.2 ± 1.9	61.5 ± 2.1	60.2 ± 2.0	48.9–57.9
	**LPS**	62.4 ± 1.8	61.7 ± 2.3	59.9 ± 0.8	59.8 ± 1.7	59.5 ± 1.7	
**MCH (pg)**	**Vehicle Control**	19.2 ± 0.7	19.8 ± 0.5	19.5 ± 0.5	19.2 ± 0.6	18.9 ± 0.6	17.1–20.4
	**LPS**	19.6 ± 0.3	19.5 ± 0.9	19.2 ± 0.2	19.4 ± 0.5	19.2 ± 0.6	
**MCHC (g/dL)**	**Vehicle Control**	31.4 ± 0.2	31.5 ± 0.6	31.8 ± 0.2	31.3 ± 0.5	31.5 ± 0.5	32.9–37.5
	**LPS**	31.4 ± 0.5	31.5 ± 0.5	32.0 ± 0.3	32.4 ± 0.3*	32.3 ± 0.3*	
**Plt (th/μL)**	**Vehicle Control**	1337.6 ± 176.4	1284.0 ± 196.8	1205.8 ± 138.1	1189.6 ± 147.6	1193.0 ± 88.0	638.0–1177.0
	**LPS**	1356.6 ± 170.3	858.6 ± 162.8*	655.8 ± 118.2*	75.8 ± 9.2*	62.4 ± 16.3*	
**Total Leukocyte (th/μL)**	**Vehicle Control**	3.75 ± 0.91	6.12 ± 0.57	3.53 ± 1.00	4.60 ± 1.28	5.26 ± 0.58	1.96–8.25
	**LPS**	2.91 ± 0.59	0.92 ± 0.26*	1.61 ± 0.86*	4.02 ± 1.80	5.81 ± 1.47	
**Neutrophil (th/μL)**	**Vehicle Control**	0.60 ± 0.14	1.68 ± 0.38	1.18 ± 0.34	0.66 ± 0.29	0.96 ± 0.27	0.22–1.57
	**LPS**	0.16 ± 0.09*	0.38 ± 0.15*	1.06 ± 0.73	2.66 ± 1.35*	1.82 ± 1.01*	
**Lymphocyte (th/μL)**	**Vehicle Control**	2.94 ± 0.78	4.04 ± 0.32	2.16 ± 0.67	3.76 ± 1.05	4.04 ± 0.71	1.41–7.11
	**LPS**	2.66 ± 0.53	0.52 ± 0.23*	0.48 ± 0.15*	1.18 ± 0.45*	3.42 ± 0.94	
**Monocyte (th/μL)**	**Vehicle Control**	0.14 ± 0.05	0.28 ± 0.08	0.10 ± 0	0.14 ± 0.05	0.18 ± 0.08	0.03–0.18
	**LPS**	0 ± 0*	0 ± 0*	0.02 ± 0.04*	0.10 ± 0.07	0.48 ± 0.15*	
**Eosinophil (th/μL)**	**Vehicle Control**	0 ± 0	0.06 ± 0.05	0.08 ± 0.04	0.06 ± 0.05	0.08 ± 0.04	0.01–0.16
	**LPS**	0 ± 0	0 ± 0*	0.08 ± 0.04	0 ± 0*	0.04 ± 0.05	
**Basophil (th/μL)**	**Vehicle Control**	0 ± 0	0 ± 0	0 ± 0	0 ± 0	0 ± 0	0–0.05
	**LPS**	0 ± 0	0 ± 0	0 ± 0	0 ± 0	0.02 ± 0.04*	

Complete cell counts of individual populations in animals from both control (saline) and LPS treated groups. n = 5 rats/time-point/group. Data were expressed as mean ± SD. MCV = mean corpuscular volume, MCH = mean corpuscular hemoglobin, MCHC = mean corpuscular hemoglobin concentration. Reference Intervals provided by Clinical Laboratories, Preclinical Services, Charles River Laboratories, Senneville, Canada, Reference Intervals for males 8–16 weeks of age.

* Indicates statistically significant difference from vehicle control P < 0.05.

Although marked decreases in circulating platelet number occurred after LPS treatment, the flow cytometric parameters of platelet activation (P-selectin expression and bound fibrinogen) showed little change over time, and no significant differences from controls at any time-point. (*Data not shown*).

### Hemostatic Pathway and Adhesion Protein Response

Mean clotting times in the APTT were significantly prolonged in LPS-treated rats compared to controls at 8 hours and 24 hours after injection (8 hour LPS mean APTT = 28.5 seconds, control = 16.9 seconds, p < 0.001; 24 hour LPS mean APTT = 23.5 seconds; control = 16.8 seconds, p < 0.001). In contrast, clotting times for the PT did not exceed the range of control rats at any time point.

**Fig 2:**
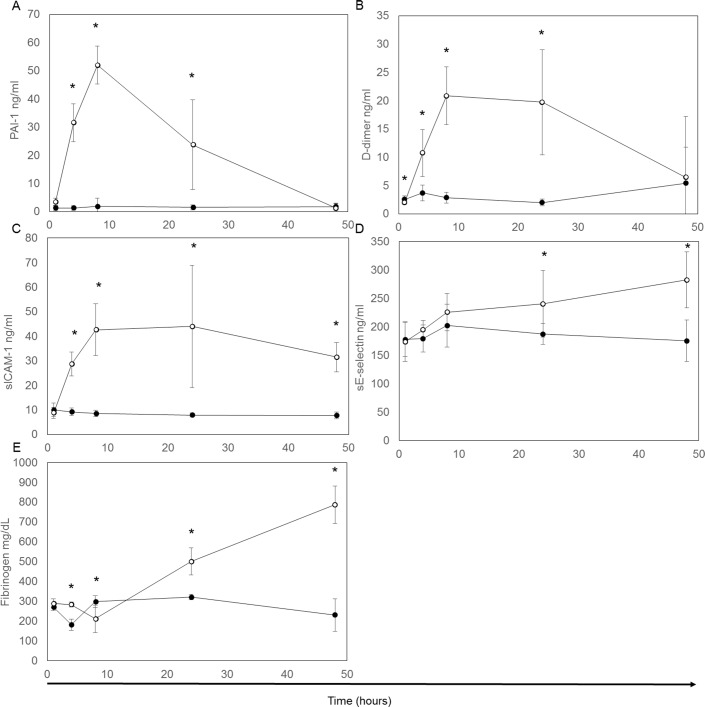
Changes in hemostatic and adhesion proteins in plasma or serum over a 48 hour period. Mean protein concentrations and standard deviation were determined in both control (●) and LPS (o) for plasma PAI-1 (A), plasma D-Dimer (B), serum sICAM-1 (C), serum sE-selectin (D), and plasma fibrinogen (F) for five animals at each time point. * indicates a significant (P<0.05) difference between the control and LPS-treated groups

Among the individual hemostatic and adhesion proteins assayed, PAI-1, D-dimer, and sICAM-1 demonstrated early increases, with increases in E-selectin and fibrinogen occurring later in the study period *(Fig 2)*. Concentrations of PAI-1 and D-dimer rose significantly at 4 hours and peaked at 8 hours to approximately 50- and 20-fold, respectively, compared to controls, before returning to baseline by 48 hours (*Fig 2A and 2B*). Concentrations of sICAM-1 in the LPS-treated rats were significantly increased by 4 hours, and remained at approximately 4.5-fold throughout 48 hours (*Fig 2C*). The concentrations of E-selectin and fibrinogen demonstrated a more gradual and progressive increase (approximately 1.6- and 2.6-fold, respectively) from 24 to 48 hours post-LPS treatment (*Fig 2D and 2E*). Due to limited sample volume, AT was only measured at 1, 4, and 8 hours, preventing complete time-course trends. We found no difference between LPS-treated rats and controls at 1 hour (mean AT = 122% LPS, control = 123%), however LPS treatment resulted in a significant decrease in AT at 4 hours that was no longer apparent at 8 hours (4 hour LPS mean AT = 111%, control = 129%, p = 0.003; 8 hour LPS mean AT = 113%, control = 118% p = 0.4)

### Plasma EV Concentration and Marker Expression

**Fig 3:**
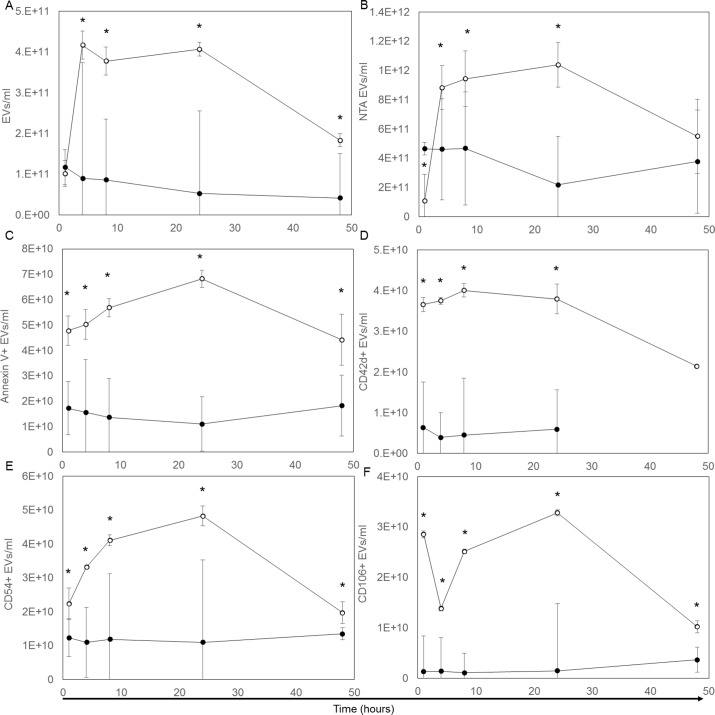
Extracellular vesicle counts/concentrations in plasma over a 48 hour period. Total EV concentrations in control (●) and LPS (o) plasma as measured by VFC. (A) Total NP concentrations in control (●) and LPS (o) plasma as measured by NTA. (B) Marker positive EV concentrations in control and LPS plasma for annexin V (C), CD42d (D), CD54 (E), and CD106 (F). Presented are means and standard deviation for five animals at each time point. * indicate a significant (P<0.05) difference between the control and LPS-treated groups.

**Fig 4:**
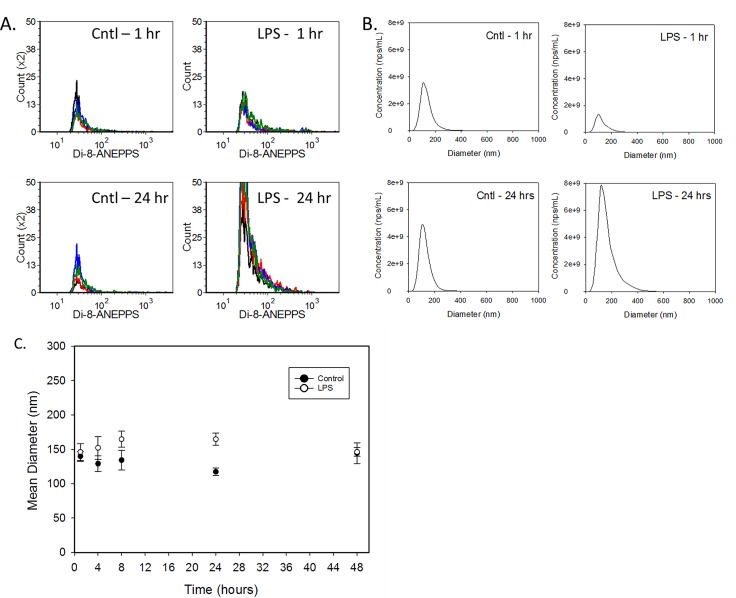
Extracellular vesicle NTA counts. Population size distributions of extracellular vesicles in rat plasma. A. Fluorescence histograms of di-8 intensity (proportional to vesicle surface area) of EVs in rat plasma 1 hour and 24 hours after treatment with vehicle or LPS. Four replicate measurements are overlaid in each panel from a single representative sample. B. NTA diameter histograms of nanoparticles in rat plasma before and 24 hours after treatment with vehicle or LPS. Each panel shows the average histogram from a single representative sample. C. NTA population mean diameter (+/- SD) of nanoparticles in rat plasma as a function of time after treatment with vehicle or LPS.

Treatment with LPS induced a rapid and marked increase in total plasma EV concentration, whereas EV concentration in the controls remained stable over the time course. Using VFC (*Fig 3A*), the EV counts for the control group were 9.43 x10^7^/μl ± 0.57 x10^7^/μl (mean ± SD for all control rats and across all time points). The EV concentration in the treatment group was significantly higher than controls by 4 hours after LPS administration, peaked at approximately 5-fold higher at 24 hours, then declined toward control levels at 48 hours. The NTA reported higher absolute nanoparticle counts for control and LPS-treated rats, but showed a similar time course of increase post-LPS treatment and return toward control levels at 48 hours (*Fig 3B*). The size distributions of the nanoparticle populations over time determined by VFC fluorescence intensity and nanoparticle diameter by NTA did not change noticeably over time for the LPS group *(Fig 4A and 4B)*, although the population mean diameters reported by NTA showed a slight increase (*Fig 4C*).

The treated rats had a rapid increase in EVs labeled with annexin V, a protein that specifically binds to outer membrane phosphatidylserine (PS), a marker of apoptosis and/or activation (*Fig 3C)*. The concentration of annexin V positive EVs in the LPS group peaked at 24 hours (mean = 6.83 x10^7^ ± 0.78 x10^7^/μl; 13% of total EVs), before declining at 48 hours. In contrast, the control rats had relatively low concentration of annexin V positive EVs throughout the study period (mean annexin V positive EVs = 1.51 x10^7^ ± 0.12 x10^7^/μl; ~11–21% of total EVs for all controls at all times).

The subset of platelet-derived EVs was identified based on CD42d expression, a constitutive component of the platelet von Willebrand factor receptor complex. The CD42d positive EV counts in LPS-treated rats rapidly increased approximately 6-fold (mean LPS group = 3.66 x 10^7^ ± 0.50 x10^7^/μl; 24% of total EVs) versus controls (mean = 5.21 x10^6^ ± 0.44 x10^6^/μl; ~4–8% of total EVs). This increase coincided with increasing annexin V positive EVs, with similar return toward control levels by 48 hours (*Fig 3D)*.

The cell adhesion proteins CD54 (ICAM-1) and CD106 (VCAM-1) were considered markers of cell activation. While counts of CD54 positive and CD106 positive EV’s increased in the LPS-treated rats, the time-course pattern varied (*Fig 3E and 3F*). After LPS treatment, the concentration of CD54 positive EVs increased steadily, reaching a peak at 24 hours (4.83 x10^7^ ± 1.21 x10^7^/μl; 8.5% of total EVs) before declining towards the relatively constant levels of the controls (1.19 x10^7^ ± 0.55 x10^7^/μl; ~8–16% of total EVs). The increase in CD106 positive EVs showed a biphasic pattern. A 20-fold increase in number was apparent at 1 hour post-treatment with LPS, followed by a transient decrease at 4 hours, an increase to peak levels at 24 hours (3.28 x10^7^ ± 0.62 x10^7^/μl; 4.9% of total EVs) before falling toward control levels at 48 hours (1.82 x10^6^ ± 0.22 x10^6^/μl; ~1–5% of total EVs) (*Fig 3F*).

Approximately two thirds of the total EV population in the controls did not label with any of the membrane antigen markers or annexin V. Lipoproteins, especially VLDL and chylomicrons may be detected in our assay due to their size similarities with microvesicles and their lipidic surfaces that may bind di-8-ANEPPS. Since total particle counts in controls did not vary over time, detection of basal shed membrane fragments or circulating lipoproteins did not preclude detection of LPS-induced increases in the number of EVs or those labeled with the activation and cell-origin markers.

### miRNA Profiling

The median total RNA yield was 5 ng/100 μL serum (range 0.75 to 50), with no apparent differences in recovery across sample timepoints or between treatment groups. Among the 378 miRNAs included on the miRNA array, 139 were excluded from analyses based on lack of primer homology with rat miRNA sequence. Several endogenous miRNAs (including mmu-miR-15b, -92a, -19b, -532-3p, and -30b) portrayed a similar, stable pattern of expression and low standard deviation (<7.6%) among all samples. Mmu-miR-15b was selected to be utilized as the endogenous reference for normalization based on its reported stability in serum [22], its observed low standard deviation in our sample set (< 4.9%), and lack of significant difference between the treatment groups, and across study time points (p≤0.05, Student’s t-test).

**Fig 5:**
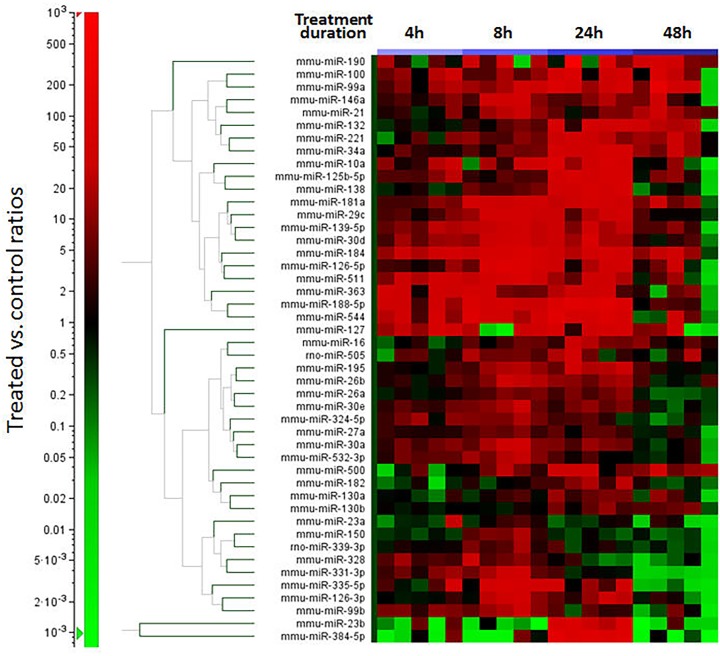
Heat Map Profiles of Serum miRNA Expression in Response to LPS Treatment. One dimensional hierarchical clustering (using Euclidian as algorithm) of 46 miRNAs significantly (p<0.05) altered in serum with a mean fold increase of >2 at more than one time point. Shown are miR-15b normalized treated vs. control ratios derived from TaqMan miRNA assays. Red illustrates increased and green decreased miRNA levels versus the control. The colored bars directly above the heat map designate the different post-LPS treatment time points and horizontal lines separate major time course profiles.

**Fig 6:**
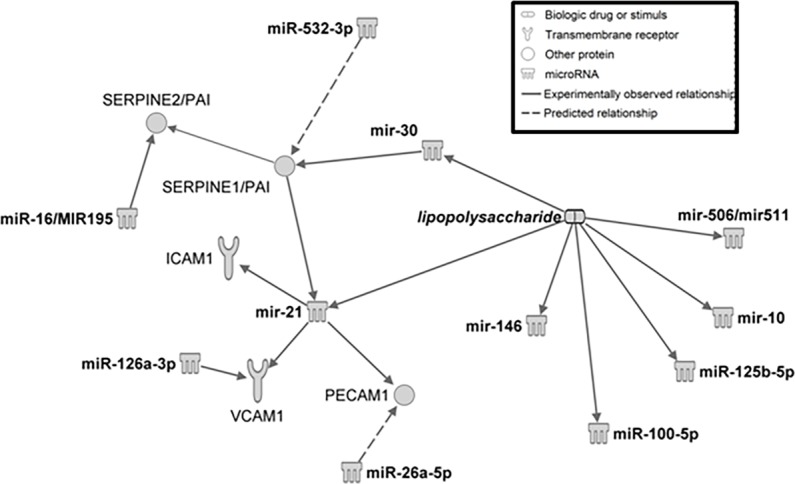
Predicted Direct Relationships Among miRNA and Hemostatic or Adhesive Protein Targets. In response to LPS stimulation, previous studies have shown increased expression of miRNAs and interaction with target proteins. The relationships depicted are generated from Ingenuity Pathway Analyses (IPA, QIAGEN Redwood city, www.quiagen.com/ingenuity). Symbol definitions are included in the figure inset.

Comparison of miRNA profiles revealed 46 miRNAs with a mean increase of two-fold or greater in the LPS group versus the time point matched controls (*Table 4)*. Most had peak increases relative to controls at the 8-hour (n = 21) or 24-hour (n = 21) post-treatment time points, with one miRNA (miR-23a) having peak increase at the 1-hour time point, one miRNA (miR-363) having a peak increase at the 4-hour time point, and 2 miRNAs (miR-21, miR-99) with peak increases at the 48 hour time point. Included among the 46 miRNAs with increased expression were 7 (miR-21, miR-16, miR-26a, miR-26b, miR-23a, miR-23b, miR-126) included in surveys of the most abundant miRNAs in human platelets [23, 24] and the miR-126 gene products miR-126-3p and miR-126-5p that are also enriched in vascular endothelial cells and endothelial microparticles [25]. Time course changes in these 46 miRNA for individual rats are displayed in a 1-dimensional heat map (*Fig 5*). Many of these miRNAs have been associated with the signaling pathways in humans for toll-like receptor 4 (TLR4), TGF-beta, and pro-inflammatory cytokines, the complement and coagulation cascades, and processes such as leukocyte migration [26]. Among the individual miRNAs previously associated with endothelial cell and monocyte activation and sepsis in humans and rodents *[27–29],* we found increases in our study of miR-16, miR-21, miR-126, miR-146a, miR-150, miR-511, and miR-23b. The adhesion and hemostatic proteins monitored in our study, sICAM-1, E-selectin, fibrinogen, and PAI-1 have been identified as targets of miRNA regulation in human cell cultures and clinical patients [29–33]. Of the 46 increased miRNA, sICAM-1 was the predicted target of 6 (miR-23b, miR-27a, miR-99a, miR-100, miR-324-5p, miR-363); PAI-1 was the predicted target of 4 (miR-30a, miR-30d, miR-182, miR-384-5p), E selectin the predicted target of 2 (miR-16; miR-195) and the alpha chain of fibrinogen the predicted target of miR-29c [26]. The IPA pathway analysis (*Fig 6*), diagrams the relationships generated from other published LPS stimulation experiments among the increased miRNA in our study and their potential hemostatic/adhesive protein targets.

**Table 4:** miRNA Changes in Serum from LPS-Treated Rats.

miRNA	1hr	4hr	8hr	24hr	48hr
mmu-miR-100	2.64	**3.02**	**2.45**	9.64	5.17
mmu-miR-10a	1.16	2.90	3.65	**13.30**	1.10
mmu-miR-125b-5p	0.33	**2.48**	1.66	**11.51**	1.62
mmu-miR-126-3p	0.82	1.02	4.71	**3.13**	0.77
mmu-miR-126-5p	1.52	1.83	**18.07**	**5.51**	1.87
mmu-miR-127	0.29	6.51	3.13	**9.30**	2.97
mmu-miR-130a	0.32	0.90	1.23	**2.38**	1.28
mmu-miR-130b	0.66	0.95	0.98	**2.02**	1.81
mmu-miR-132	0.69	0.92	1.78	**7.87**	3.52
mmu-miR-138	0.48	0.89	**2.05**	**10.29**	0.63
mmu-miR-139-5p	0.92	**2.96**	**5.40**	**4.33**	1.65
mmu-miR-146a	1.17	1.88	**5.10**	**2.59**	2.11
mmu-miR-150	0.60	0.69	**2.45**	0.71	0.50
mmu-miR-16	0.37	0.82	1.84	**2.99**	1.73
mmu-miR-181a	1.15	**2.09**	**7.09**	**6.88**	2.70
mmu-miR-182	0.37	0.60	1.13	**2.82**	0.73
mmu-miR-184	0.07	**5.39**	**13.54**	**7.88**	3.81
mmu-miR-188-5p	0.04	**4.80**	**10.35**	**32.91**	1.34
mmu-miR-190	0.03	1.70	5.48	103.93	**6.28**
mmu-miR-195	0.14	1.22	**3.12**	**4.35**	1.22
mmu-miR-21	0.77	1.45	**3.30**	1.94	**3.37**
mmu-miR-221	2.15	1.36	**2.36**	**4.78**	**3.25**
mmu-miR-23a	**3.23**	1.39	1.29	0.82	0.61
mmu-miR-23b	0.66	0.94	0.29	**13.45**	0.45
mmu-miR-26a	0.84	0.99	**2.22**	**2.28**	0.58
mmu-miR-26b	1.41	1.39	**3.70**	**3.01**	1.21
mmu-miR-27a	0.81	1.36	**2.09**	**1.75**	0.84
mmu-miR-29c	0.52	1.87	**7.06**	**4.80**	1.14
mmu-miR-30a	0.84	**2.11**	**4.00**	**2.49**	0.83
mmu-miR-30d	0.95	**2.71**	**5.90**	6.82	1.15
mmu-miR-30e	0.20	1.76	**3.28**	**2.31**	0.87
mmu-miR-324-5p	0.58	2.23	**2.83**	1.51	0.71
mmu-miR-328	1.86	**2.06**	**2.74**	0.81	0.40
mmu-miR-331-3p	0.31	**2.23**	**4.16**	1.06	0.15
mmu-miR-335-5p	0.35	3.20	**8.23**	4.13	0.21
mmu-miR-34a	0.06	1.41	**2.09**	**10.20**	**2.95**
mmu-miR-363	0.08	**5.54**	5.30	**4.96**	1.33
mmu-miR-384-5p	2.62	0.72	0.67	**32.28**	0.31
mmu-miR-500	0.59	0.89	2.29	**4.02**	**3.72**
mmu-miR-511	**2.78**	**5.52**	**10.72**	**4.29**	1.35
mmu-miR-532-3p	1.11	1.97	**3.38**	1.38	0.84
mmu-miR-544	0.07	5.77	11.99	**21.49**	1.79
mmu-miR-99a	4.14	**2.56**	**4.42**	11.22	20.52
mmu-miR-99b	1.35	**2.40**	**3.39**	1.49	0.87
rno-miR-339-3p	0.75	1.06	**2.23**	1.15	0.54
rno-miR-505	1.07	1.28	**2.55**	3.39	1.56

miRNA RQ values normalized to miR-15b and calibrated to mean of time-matched control rats dosed with vehicle. Values highlighted and bold met the cutoff criteria of a 2-fold increase over time-matched control values and a p-value < 0.05 using a paired student’s t-test, assuming equal variance.

### Gross Anatomic and Microscopic Pathology

No gross or microscopic abnormalities were noted in the controls. The gross anatomic changes attributed to LPS–induced inflammation included small thymus size, at 24 and 48 hours, and discoloration and abnormal consistency of the intestinal contents.

**Fig 7:**
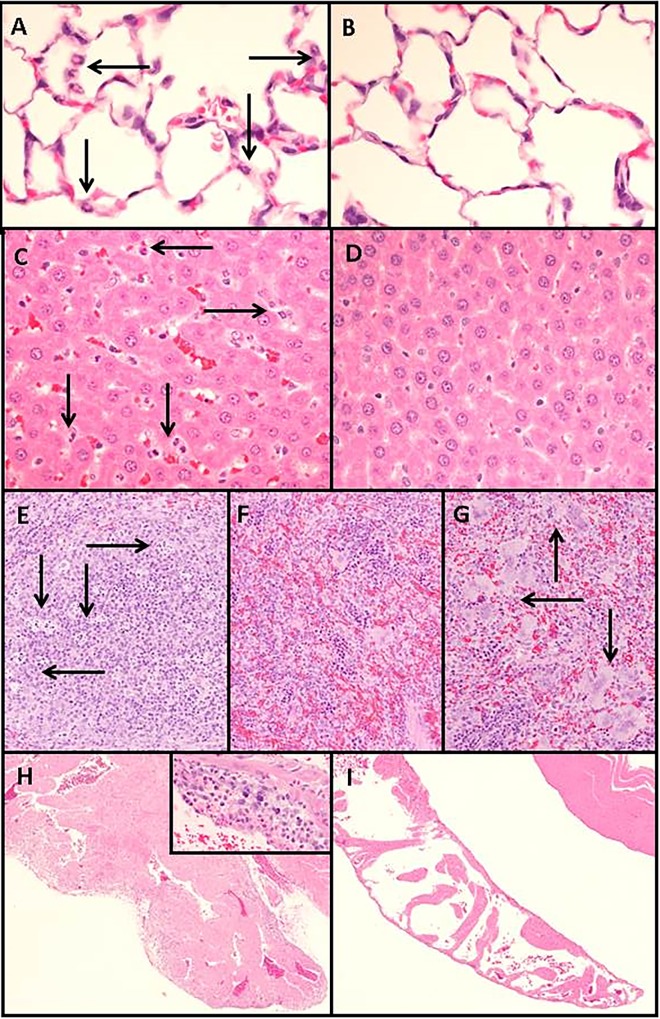
Histopathology, hematoxylin and eosin-stained sections. (A) Neutrophil sequestration (arrows) within pulmonary alveolar capillaries typical of 5/5 rats at hours 4, 8, 24, and 48 post-intraperitoneal LPS injection. (B) Normal pulmonary alveolar capillaries at 48 hours post-intraperitoneal saline injection. (C) Neutrophil sequestration (arrows) within hepatic sinusoids typical of 5/5 rats at hour 4 and 8 post-intraperitoneal LPS injection. (D) Normal hepatic sinusoids at 8 hours post-intraperitoneal saline injection. 400 X original magnification A, B, C, and D. (E) Single cell necrosis of lymphocytes (arrows) within splenic lymphoid follicles typical of 5/5 rats at hours 4 and 8 post-intraperitoneal LPS injection. (F) Normal spleen at 8 hours post-intraperitoneal saline injection. (G) Increased megakaryocytes (arrows) within spleen typical of 5/5 rats at hour 48 post-intraperitoneal LPS injection. 200 X original magnification E, F, and G. (H) Leukocyte accumulation in subepicardial myocardium of the right atrium typical of 5/5 rats at hour 48 post-intraperitoneal LPS injection. Inset 400X original magnification. (I) Normal right atrium at 48 hours post-intraperitoneal saline injection. 40 X original magnification H, I.

Microscopic abnormalities were absent at the 1-hour time point, however sequestration of neutrophils in lung and liver was found in all LPS-treated rats at the 4-hour and subsequent time points (*Fig 7*). The earliest evidence of inflammation included a slight accumulation of neutrophils in the pulmonary alveolar capillaries and hepatic sinusoids at the 4-hour time point (*Fig 7A and 7C*). Pulmonary sequestration of neutrophils was then consistently found through the remaining time points. Minimal to slight single-cell necrosis of splenic lymphocytes developed in the 8 and 24-hour timepoints (*Fig 7E*). At 48 hours, all LPS-treated rats had minimal to slight periportal hepatocellular vacuolation, splenic megakaryocytosis, and accumulations of leukocytes within the epicardium and myocardium (*Fig 7G and 7H)*. One rat had evidence of thrombosis, limited to a single fibrin embolus in the lumen of a small muscular pulmonary artery in which there was also neutrophil sequestration. No microscopic abnormalities were found in the brain, kidney, or mesentery in the LPS-treated rats.

## Discussion

In this model, LPS triggered acute inflammation and achieved non-lethal alterations in hemostasis defined by three overlapping time course phases of the host response. The first phase (0 to 8 hour) was characterized by early leukocyte mobilization. In the second phase (8 to 24 hour), endothelial cell and platelet activation, procoagulant imbalance, intrapulmonary and intrahepatic sequestration of neutrophils, and peak increases in circulating miRNA occurred. In the third phase (24 to 48 hours), we found evidence of recovery, rather than progression to overt DIC. By 48 hours, the early, marked increases in circulating PAI-1, sICAM-1, D dimer, and procoagulant EVs either returned to, or fell toward baseline and the microvascular thrombi characteristic of LPS-induced DIC were not apparent at necropsy. The ultimate survival of all LPS-treated rats without succumbing to DIC provides evidence of a systemic mechanism for restraining coagulation in a pro-inflammatory environment.

The systemic inflammatory response to LPS is driven by leukocyte activation and cytokine release [5–7]. The accessory proteins, lipoprotein binding protein (LBP) and CD14, interact with LPS and then engage the MD2/TLR4 membrane receptor complex which in turn initiates signaling through TLR4, translocation of transcription factors to the nucleus, and upregulation of inflammatory cytokine synthesis [34]. Dramatic increases in circulating cytokines develop within 1 to 3 hours of LPS administration. In concert with cytokine release, activated monocytes express tissue factor, capable of triggering thrombin generation in the vascular space through interaction with Factor VIIa and assembly of coagulation complexes on membranes that express PS [4, 8]. In addition to the secondary effects of cytokine and thrombin-mediated protease activated receptor stimulation on platelets and endothelial cells, LPS directly initiates signaling through the TLR4/MYD88 pathway to activate both these cell-types [5]. This complexity of response complicates identification of features of inflammation that dictate progression to overt DIC versus mediators of a compensated, antithrombotic milieu that protects blood flow [10, 35].

In our study, intraperitoneal LPS induced inflammatory cell margination within 1 hour, with marked decreases in circulating neutrophil and monocyte counts at this earliest time point. Recent studies reveal that directional neutrophil migration is orchestrated by the simultaneous interaction of neutrophils with activated vascular endothelium and degranulated platelets [36]. Due to their membrane expression of molecules with functional and signaling activities, EVs are under investigation as mediators of such coordinated systemic responses [37, 38]. In our study, the systemic concentration of EVs expressing PS, platelet, or cell-adhesion markers rose by 1 hour, coincident with the earliest changes in leukocyte count and before any decreases in platelet count (*Figs 1–3*). Moreover, high counts of circulating EVs persisted with little histologic evidence of tissue injury or necrosis, suggesting that EVs reflected cell activation and regulatory response rather than membrane vesiculation associated with widespread cell death.

In concert with high levels of procoagulant EVs, we found evidence of excess thrombin formation and dysregulated coagulation occurring maximally at 8 hours after LPS administration. Activated endothelial cells promote thrombin generation while losing their ability to inhibit coagulation through downregulation of thrombomodulin and impaired formation of activated protein C [8, 35]. At the 8 hour time point, the LPS-treated rats had prolonged in vitro clotting times, peak increase in circulating D-dimers, a marker of thrombin-mediated fibrin cross-linkage, and peak levels of PAI-1, a potent fibrinolysis inhibitor. This pattern of abnormalities at 8 hours was also found in a previous study of DIC in rats induced by a two-fold higher dose of LPS [6]. In contrast to the transient changes in our sub-lethal model, most of the rats in that study succumbed to multi-organ failure between 8 and 24 hours after LPS treatment. Histologic examination in that study revealed microvascular thrombosis, necrosis, and hemorrhage, the pathognomonic findings of overt DIC, as the cause of death [6].

One of the regulatory roles proposed for circulating EVs are carriers of miRNAs, small noncoding RNA molecules that target messenger RNA for degradation or translational repression [39]. We found that a subset of 46 circulating miRNA increased in response to LPS, with peak levels during the 8 to 24 hour interval followed by decrease toward baseline by 48 hours. Among these, miR-150, miR-23b, and miR-146a have been previously identified as potential circulating mediators or biomarkers of inflammatory pathways in sepsis. An *in vivo* study of LPS administration to rats described amelioration of inflammation in response to a 20-hydroxyeicosatetraenoic acid analog in concert with decreases in miR-150 [40]

In a recent review of miRNA in septic patients, changes in circulating levels of miR-150 and miR-146a were common findings, however any consistent association with clinical outcome remains ill-defined [27]. As depicted in Fig 6, among the miRNAs elevated in serum in our study, LPS has been shown to increase expression of miR-10a, miR-100, miR-508/511, miR-30c, and miR-125b in human fibroblast-like synoviocytes [41], increase expression of miR-146a in a human monocyte cell line [42], and increase miR-21 in cultured murine monocytes [43]. The physiologic or pathophysiologic effects of these increases, however, are not defined and contradictory results are obtained in different cell systems. For example, miR-21 overexpression in the murine monocytes attenuated cytokine-induced inflammation and lipid accumulation, whereas upregulation of miR-21 in human endothelial cells enhanced the inflammatory response [44]. High plasma PAI-1 induced by LPS administration or sepsis is typically associated with intravascular fibrin deposition due to delayed fibrinolysis [4, 6, 8, 10]. Paradoxically, enhanced muscular fibrosis accompanies decreased expression of PAI-1 attributed to miR-21 inhibition in an engineered murine model of muscular dystrophy [45]. In this model, the lack of PA1-1 inhibition results in urokinase-mediated activation of TGF beta and the downstream effects of enhanced fibroblast proliferation and collagen deposition. Defining potential regulatory roles of circulating miRNA on hemostatic pathways will clearly require *in vivo* model systems to complement expression studies based on cell culture experiments.

Our study’s limitations include low numbers of subjects and use of a single LPS dose that precluded comparisons across a dosage range. Further characterization of the EVs and miRNA species was hampered by limited availability of defined rat cell-origin markers and few miRNA in the commercial rodent array targeting procoagulant proteins or defined platelet activation pathways. While the time course of increases in procoagulant EVs and subsets of circulating miRNA suggest a potential role in promoting and controlling the hemostatic response to inflammatory insult, our study is not designed to differentiate epiphenomenon from causal factors.

Our adaptation of an LPS treatment protocol is relevant for preclinical studies of inflammation-induced coagulation and the protective response that restores hemostatic balance. Although beyond the scope of this pilot project, the time course of response we identified will facilitate expression analyses of putative miRNA target genes. We identified rapid, but transient, increases in circulating concentrations of sICAM-1, PAI-1, and D-dimer denoting early cellular activation and dysregulated coagulation and fibrinolysis that promote thrombus formation. Unlike observational studies of circulating EVs and miRNAs in clinical patients with heterogeneous disease states, rat models allow more rigorous experimental controls to differentiate biomarker profiles representing effective versus ineffective compensatory response to defined inflammatory stimuli. Ultimately, bridging the gap in the preclinical setting holds promise for more effective translational biomarker panels to predict and prevent thrombotic complications in clinical practice.
